# Draft Genome Sequence of *Serratia marcescens* Strain LCT-SM166, a Space Flight Strain with a Specific Carbon Source Utilization Pattern

**DOI:** 10.1128/genomeA.00069-14

**Published:** 2014-02-13

**Authors:** Yajuan Wang, Yu Du, Yanting Yuan, Yinghua Guo, Junfeng Wang, Tianzhi Li, De Chang, Yan Liu, Xuege Jiang, Wenkui Dai, Changting Liu

**Affiliations:** a Nanlou Respiratory Diseases Department, Chinese PLA General Hospital, Beijing, China; b BGI-Shenzhen, Shenzhen, China

## Abstract

*Serratia marcescens* has been detected in space habitats. To explore the influence of the space flight environment on this bacterium, we investigated the genome sequence of LCT-SM166, which was isolated after space flight and has a specific carbon source utilization pattern.

## GENOME ANNOUNCEMENT

There is significant evidence that changes in bacterial properties are associated with the space flight environment. These changes include alterations in proliferation rate, cell physiology, cell metabolism, biofilm formation, virulence, and drug resistance (1). *Serratia marcescens* appears to be a common environmental organism and has also been detected in European spacecraft-associated clean rooms and in the Herschel Space Observatory (2). Recently, *S. marcescens* was recognized as a prominent opportunistic pathogen associated with numerous outbreaks and opportunistic infections (3). To investigate the response of *S. marcescens* to a space environment, *S. marcescens* CGMCC 1.1857, obtained from the China General Microbiological Culture Collection Center (CGMCC), was carried into space by the Shenzhou VIII spacecraft for 398 h. Strain LCT-SM166 was selected after the space flight because it has apparent differences in carbon source utilization from those of the ground control strain; this strain was subjected to whole-genome sequencing.

The genome of *S. marcescens* LCT-SM166 was sequenced with an Illumina HiSeq 2000 instrument according to the manufacturer’s instructions. A high-molecular-weight genomic DNA sample from *S. marcescens* LCT-SM166 was used to construct 500-bp and 6,000-bp random sequencing libraries. The read length was 90 bp, and 936 Mbp and 925 Mbp were produced for the 500-bp and 6,000-bp libraries, respectively. The filtered high-quality reads were assembled into 47 contigs in 13 scaffolds using SOAP*denovo* version 1.06. The scaffold N_50_ was determined to be 1,523,938 bp, and the N_90_ was determined to be 1,415,039 bp. The longest scaffold is 2,081,004 bp. The full length of the assembly is 5,070,021 bp, with a G+C content of 59.72%.

Finally, based on the assembled sequence, 4,781 coding sequences (CDSs) with an average gene length of 927 bp were predicted using Glimmer version 3.02. The genes were annotated by BLAST analysis of the KEGG, COG, Swiss-Prot, TrEMBL, and NR public databases for functional annotation. There were 3,731 CDSs identified in the KEGG database and 4,362 CDSs in the NR database. In addition, there were 26 rRNAs predicted using RNAmmer and 71 tRNAs predicted using tRNAscan-SE. We found 2,420 transposons using RepeatMasker and RepeatProteinMasker. Finally, there were 6,137 tandem repeats predicted using TRF software, which comprised 0.62% of the total assembly.

### Nucleotide sequence accession number.

This whole-genome shotgun project of *S. marcescens* LCT-SM166 has been deposited at DDBJ/EMBL/GenBank under the accession no. ATJW00000000. The version described in this paper is the first version.
